# The Modified Heidelberg and the AI Appendicitis Score Are Superior to Current Scores in Predicting Appendicitis in Children: A Two-Center Cohort Study

**DOI:** 10.3389/fped.2020.592892

**Published:** 2020-11-17

**Authors:** Carolin Stiel, Julia Elrod, Michaela Klinke, Jochen Herrmann, Carl-Martin Junge, Tarik Ghadban, Konrad Reinshagen, Michael Boettcher

**Affiliations:** ^1^Department of Pediatric Surgery, University Medical Center Hamburg-Eppendorf, Hamburg, Germany; ^2^Section of Pediatric Radiology, Department of Diagnostic and Interventional Radiology and Nuclear Medicine, University Medical Center Hamburg-Eppendorf, Hamburg, Germany; ^3^Department of Pediatric Radiology, Altonaer Kinderkrankenhaus, Hamburg, Germany; ^4^Department of General, Visceral and Thoracic Surgery, University Medical Center Hamburg-Eppendorf, Hamburg, Germany

**Keywords:** appendicitis, children, diagnosis, predicition, scores

## Abstract

**Background:** Acute appendicitis represents the most frequent reason for abdominal surgery in children. Since diagnosis can be challenging various scoring systems have been published. The aim of this study was to evaluate and validate (and improve) different appendicitis scores in a very large cohort of children with abdominal pain.

**Methods:** Retrospective analysis of all children that have been hospitalized due to suspected appendicitis at the Pediatric Surgery Department of the Altonaer Children's Hospital and University Medical Center Hamburg-Eppendorf from 01/2018 until 11/2019. Four different appendicitis scores (Heidelberg Appendicitis Score, Alvarado Score, Pediatric Appendicitis Score and Tzanakis Score) were applied to all data sets. Furthermore, the best score was improved and artificial intelligence (AI) was applied and compare the current scores.

**Results:** In 23 months, 463 patients were included in the study. Of those 348 (75.2%) were operated for suspected appendicitis and in 336 (96.6%) patients the diagnosis was confirmed histopathologically. The best predictors of appendicitis (simple and perforated) were rebound tenderness, cough/hopping tenderness, ultrasound, and laboratory results. After modifying the HAS, it provided excellent results for simple (PPV 95.0%, NPV 70.0%) and very good for perforated appendicitis (PPV 34.4%, NPV 93.8%), outperforming all other appendicitis score.

**Discussion:** The modified HAS and the AI score show excellent predictive capabilities and may be used to identify most cases of appendicitis and more important to rule out perforated appendicitis. The new scores outperform all other scores and are simple to apply. The modified HAS comprises five features that can all be assessed in the emergency department as opposed to current scores that are relatively complex to utilize in a clinical setting as they include of up to eight features with various weighting factors. In conclusion, the modified HAS and the AI score may be used to identify children with appendicitis, yet prospective studies to validate our findings in a large mutli-center cohorts are needed.

## Background

Abdominal pain is one of the most common reasons for pediatric emergency presentation and acute appendicitis represents the most frequent reason for abdominal surgery in children (1). The incidence of appendicitis is reported to be 151/100,000 person-years in Western Europe (2). Diagnosis can be very challenging, particularly in the early stages of appendicitis when clinical manifestations may be less typical and is particularly demanding in younger children (3). The reported negative appendectomy rate in patients with clinically diagnosed suspected acute appendicitis is about 15–20% and can be associated with considerable morbidity (4). Rapid diagnosis is important, because increased time between onset of symptoms and surgical intervention is associated with increased risk of appendiceal perforation and therefore with increased morbidity (5).

Thus, different elements of history, clinical examination, as well as laboratory and radiologic findings are used to diagnose appendicitis. In order to further improve diagnosis various algorithms and scoring systems have been developed. The most widely used scores are the Heidelberg Appendicitis Score (HAS), the Pediatric Appendicitis Score (PAS), the Alvarado-Score and the Tzanakis-Score (6–9). While both the HAS and the PAS are designed for pediatric patients (7, 9), the Alvarado- and the Tzanakis-Score were conceptualized in a broader patient population (6, 8). The HAS appears to be the simplest to apply, but has not been validated other than in its initial cohort. Even though the different scores are suitable to predict appendicitis, their practicability is limited due to the multitude of different features. The clinician has to access up to eight factors, which are weighted unequally, making them difficult to use in an emergency department setting. The aim of this current study was to evaluate and validate the different scoring systems in two large pediatric surgery centers.

## Materials and Methods

### Study Cohort Characteristic

Retrospective cohort study of all children (age 1–17) that were hospitalized for suspected appendicitis as assumed by a triage nurse at the department of Pediatric Surgery at the Altonaer Children's Hospital in Hamburg (AKK) and the University Medical Center Hamburg-Eppendorf (UKE) between January 2018 and November 2019. The nurses direct the patients either to the surgical or the pediatric department in the interdisciplinary emergency department. The study is a sub-analysis of two prospective cohort studies that are in accordance with the guidelines of the Medical Research Ethics Committee of Hamburg (Ethik-Kommission der Ärztekammer Hamburg, PV5459, and PV5891).

### Study Cohort Outcomes

Patients were selected from the hospital database by ICD-10-Codes for abdominal pain, appendicitis and functional intestinal disorder. Children with chronic medical conditions were excluded (i.e., chronic constipation and Hirschsprung's disease). Medical files, including patient charts, operating theater records, and office notes, were reviewed and routinely obtained characteristics were recorded. These included demographic data (age and sex), clinical history (duration of abdominal pain, nausea/vomitus, stool consistency, dysuria, and pyrexia), physical signs (tenderness right lower quadrant, rebound tenderness, cough/hopping tenderness in the right lower quadrant, and psoas sign), laboratory results (white cell counts (WBC) >11 × 10^9^/L, C-reactive protein (CRP) >20 mg/L, neutrophilia >75% respectively >7.9 × 10^9^/L), urine-analysis (nitrite, ketone, and leucocytes), ultrasound findings (appendix outer diameter > 6 mm, surrounding tissue involvement, appendix wall hyper-perfusion, free fluids, wall edema, and signs of constipation), intraoperative findings and histopathology of the appendix. All items listed were evaluated and utilized for the scores. Physical examinations were performed by a resident and/or a senior physician of pediatric surgery and ultrasound examinations were performed by a resident of Pediatric Surgery or Pediatric Radiology. All removed appendices underwent a standardized histopathological examination at the same pathology. The differentiation of distinct stages of appendicitis was based on histopathological findings, which is usual practice in studies of appendicitis (10). The assessment of perforation was based on the operating theater records. Four different appendicitis scores including the HAS (7), the Alvarado score (6), the PAS (9) and the Tzanakis (8) score were assessed in all children (compare Table 1).

**Table 1:** Display of the different scoring systems for diagnosing appendicitis, including the different weighing factors for each score.

	**Alvarado**	**PAS**	**Tzanakis**	**HAS**	**Mod**	**AI**
	**(6)**	**(9)**	**(8)**	**(7)**	**HAS**	**Score**
Tenderness right lower quadrant	2	2	4	1	1	
Rebound tenderness	1		3	1	1	1
WBC (>12 × 10^9^/l/>11 × 10^9^/l)	2	1	2		1	1
CRP (>20 mg/L)					1	1
US demonstrating APP			6	1	1	1
Continuous pain				1		
Cough/hopping tenderness		2				
Nausea/vomiting	1	1				
Anorexia/urine acetone	1	1				
Migration of pain	1	1				
Temperature (>38.5°C)	1	1				
Neutrophilia (>7.9 × 10^9^/l/>75%)	1	1				
Positive score	5/10	6/10	8/15	3/4	3/5	2/4

*US demonstrating appendicitis comprises the appendix diameter > 6 mm and/or signs of inflammation such as wall edema, hyperemia, and surrounding inflammation. WBC, white blood count; CRP, C-reactive protein; US, ultrasound; APP, appendicitis*.

### Development of the Scores

In addition to current scores, a modified Heidelberg Appendicitis Score (mHAS) based on a classification and regression tree (CART) analysis and an AI based score were developed. Statistics were performed using SPSS Statistics 26 (IBM, NY, USA) and R 4.0 (Foundation for Statistical Computing, Vienna, Austria.

The HAS was improved using CART analysis, resulting in the “modified HAS.” Classification and regression tree has some advantages over logistic regression analysis such as the ability to utilize large numbers of predictor variables and non-reliance on the underlying distributions for statistical inference (11). For validation, data was randomly divided into two parts and 70% of the data were used for training and 30% for testing. To avoid over-fitting the decision tree was pruned for a small risk value.

Ultimately, the AI-based approach was calculated using the R package Random Forest (12). Random Forest is a method of regression which can capture non-linear relationships by averaging the prediction of multiple decision trees (13). For validation, the model was trained by randomly splitting the entire data into two parts, where 70% of the data were used for training and 30% of the data for testing. This was performed 20 times with new random distribution of the data, to eliminate outliers, and the average of the results were taken. In order to prevent over-fitting of the model to the data of the current study which could limit generalization in future real-world use, the random forest method comprises the use of different trees of which each tree is trained on a different bootstrapped dataset. In our case 200 trees were used.

For both analysis (AI and modified HAS) all features listed in the methods section were utilized. Data is presented as mean (standard deviation). The level of significance was set to 0.05.

## Results

In total 550 patients were included in the current study. After exclusion of children with chronic medical conditions, 463 patients fulfilled the inclusion criteria. Of those, 348/463 were operated for suspected appendicitis and in 336/348 of these patients, appendicitis was confirmed histopathologically. The negative appendectomy rate was thus 12/348 (3.4%). In 234/348 (67.2%) children appendicitis was simple (phlegmonous and gangrenous) and 102/348 (29.3%) children had perforated appendicitis. Children without appendicitis (127/463) suffered from constipation, mesenteric lymphadenitis, gastroenteritis, unspecific abdominal pain, ovarian cysts, or adnexitis. Diagnosis was confirmed by observation.

Mean age of the cohort was 10.9 (3.7) years and 264/463 (57.0%) were female. Children with simple appendicitis were significantly younger than patients with perforated appendicitis [simple 10.9 (3.3) vs. 10.0 (4.2), *p* = 0.038]. Children without appendicitis were significantly older than children with appendicitis [non-appendicitis 11.6 (3.7) vs. appendicitis 10.6 (3.6) years, *p* = 0.006]. Gender distribution was similar in patients with appendicitis (simple 143/234 vs. perforated 70/102 female, *p* = 0.19). In the group without appendicitis, significantly less subjects were female (non-appendicitis 53/127 vs. appendicitis 212/336 female, *p* < 0.0001). All children presented with right sided abdominal pain. The duration of the symptom varied significantly between appendicitis compared to non-appendicitis [39.6 (43.3) vs. 57.8 (73.4) h, *p* = 0.001]. Children with simple appendicitis had a significantly shorter duration of symptoms [52.1 (48.7) vs. 34.3 (39.7) h, *p* = 0.001].

The clinical feature with the highest predictive value of the patient's history was anorexia; if presented it had high positive predictive values (Table 2). Clinical signs such as tenderness in the right lower quadrant, rebound tenderness, and cough/hopping tenderness had excellent predictive values (Table 2). WBC, CRP, neutrophils, and urine ketones were considerably elevated in most, but not all children with appendicitis (Table 2). Moreover, most children with appendicitis had pathological ultrasound findings (Table 2).

**Table 2:** Positive predictive value (PPV) and negative predictive value (NPV) to diagnose appendicitis (selection of the items that were used in the evaluated appendicitis scores) in the entire cohort of children with abdominal pain.

	**Present**	**PPV % (CI 95%)**	**NPV % (CI 95%)**
Continuous pain	7/463	40.0 (8.0–79.9)	50.0 (34.0–69.9)
Nausea/vomiting	319/463	81.5 (78.4–84.5)	45.3 (38.7–51.7)
Anorexia	176/463	82.1 (76.2–87.4)	38.0 (32.0–43.4)
Migration of pain	0/463	/	/
Temperature (>38.5°C)	312/463	97.4 (85.0–99.9)	30.0 (28.8–30.3)
Tenderness RLQ	334/463	73.3 (72.5–73.9)	58.3 (28.9–83.4)
Rebound tenderness	293/463	89.7 (85.3–93.2)	36.5 (32.4–39.8)
Cough/hopping tenderness	205/463	89.7 (86.0–93.0)	37.2 (29.0–44.5)
Leukocytosis (>11 mrd/L)	333/463	85.7 (82.4–88.7)	48.8 (43.3–53.9)
Neutrophilia (>7.9 mrd/L or >75%)	72/463	96.4 (92.5–99.3)	13.6 (4.0–21.1)
CRP (>20 mg/L)	332/456	94.6 (91.1–96.9)	47.7 (44.4–49.9)
US demonstrating APP	287/456	95.9 (93.0–97.8)	68.1 (63.8–70.9)

*RLQ, right lower quadrant; CRP, C-reactive protein; US, ultrasound; APP, appendicitis*.

Clinical data was available in all patients. Laboratory and ultrasound were available in 456/463 patients. For the scores only patients with complete data samples (456/463 patients) were included. Applying the current scores revealed low to moderate sensitivity (31.0–80.4%) with moderate to high specificity (78.7–96.1%) for simple appendicitis and a slightly improved sensitivity (40.2–84.3%) with low to moderate specificity (20.1–73.5%) for perforated appendicitis (Table 3). Thus, a CART analysis was performed and five factors quite similar to those included in the HAS were identified. In the modified HAS, pain quality, which had been present only in 7/463 patients, was “replaced” by WBC and CRP. It consists of US demonstrating APP (importance 0.18), CRP > 20 mg/L (importance 0.08), rebound tenderness (importance 0.01), WBC > 11 × 10^9^/L (importance 0.006), tenderness in the right lower quadrant (importance 0.001). The features importance indicates what features contribute most to the decision making in the model.

**Table 3:** Predictive capabilities of the current and the new score in predicting appendicitis in the entire cohort of children with abdominal pain.

	**Sensitivity (CI 95%)**	**Specificity (CI 95%)**	**PPV (CI 95%)**	**NPV (CI 95%)**	**LR+ (CI 95%)**	**LR– (CI 95%)**	**AUC (CI 95%)**
Alvarado	67.9 (65.1–70.1)	78.7 (71.6–84.8)	89.4 (85.8–92.4)	48.1 (43.7–51.8)	3.19 (2.29–4.60)	0.41 (0.35–0.49)	0.79 (0.75–0.85)
Pediatric	34.2 (32.2–35.3)	95.3 (90.0–98.0)	95.0 (89.5–97.9)	35.4 (33.4–36.4)	7.24 (3.24–18.01)	0.69 (0.66–0.75)	0.81 (0.77–0.85)
Tzanakis	80.4 (78.0–82.1)	87.4 (81.1–92.1)	94.4 (91.6–96.5)	62.7 (58.2–66.1)	6.38 (4.13–10.40)	0.23 (0.19–0.27)	0.87 (0.84–0.91)
HAS	31.0 (29.1–31.9)	96.1 (91.0–98.5)	95.4 (89.6–98.3)	34.5 (32.7–35.3)	7.86 (3.34–21.64)	0.72 (0.69–0.78)	0.84 (0.80–0.88)
Mod HAS	86.6 (84.0–89.5)	70.9 (64.0–76.9)	88.7 (86.0–91.1)	66.7 (60.2–72.4)	7.31 (4.72–11.98)	0.15 (0.13–0.19)	0.92 (0.89–0.95)
AI score	87.2 (84.6–89.5)	70.1 (63.2–76.1)	88.5 (85.9–90.8)	67.4 (60.8–73.2)	2.91 (2.30–3.75)	0.18 (0.14–0.24)	0.86 (0.82–0.90)

*The modified Heidelberg Appendicitis Score (HAS) and the Artificial Intelligence (AI) score have excellent predictive capabilities (sensitivity, positive and negative LR, and AUC) for appendicitis in children. PPV, positive predictive value; NPV, negative predictive value; LR+, positive likelihood ratio; LR–L negative likelihood ratio; AUC, area under the curve*.

Conversely, the AI based approach resulted in four factors only, interestingly unlike all other scores, tenderness of the right lower quadrant did not seem to be a decisive determinant of an acute appendicitis using this method. It consists of US demonstrating APP (importance 0.37), CRP > 20 mg/L (importance 0.25), rebound tenderness (0.21), and WBC > 11 × 10^9^/L (importance 0.08).

Both new scores achieved excellent diagnostic values for simple appendicitis and identified almost all cases of perforated appendicitis (Tables 3, 4). If only children with a positive AI score had an appendectomy, only 2/102 (2.0%) children with perforated appendicitis would have been missed, and 3/102 (2.9%) children if the modified HAS would have been applied. In contrast, the Alvarado score would have missed 16/102 (15.7%), the PAS 61/102 (59.8%) the Tzanakis score 19/102 (18.6%) and the original HAS 60/102 (58.8%). Both new scores also yield a decent specificity (modified HAS: 70.9%, AI Score: 70.1%), albeit the other scores are superior in this regard. Given the excellent sensitivity, the new scores outperform all currently available ones (Tables 3, 4).

**Table 4:** Predictive values for perforated vs. non-perforated appendicitis.

	**Sensitivity (CI 95%)**	**Specificity (CI 95%)**	**PPV (CI 95%)**	**NPV (CI 95%)**	**LR+ (CI 95%)**	**LR– (CI 95%)**	**AUC (CI 95%)**
Alvarado	84.3 (76.7–90.2)	39.3 (36.0–41.9)	37.7 (34.3–40.3)	85.2 (78.0–90.7)	1.39 (1.20–1.55)	0.40 (0.23–0.65)	0.69 (0.54–0.67)
Pediatric	40.2 (32.1–48.5)	68.4 (64.8–72.0)	35.7 (28.5–43.0)	72.4 (68.7–76.2)	1.27 (0.91–1.73)	0.87 (0.72–1.05)	0.61 (0.54–0.67)
Tzanakis	81.4 (74.2–87.5)	20.1 (16.9–22.8)	30.7 (28.0–33.1)	71.2 (60.1–80.8)	1.02 (0.89–1.13)	0.93 (0.55–1.53)	0.51 (0.44–0.58)
HAS	41.2 (33.1–49.3)	73.5 (70.0–77.0)	40.4 (32.5–48.4)	74.1 (70.6–77.7)	1.55 (1.10–2.15)	0.80 (0.65–0.96)	0.58 (0.51–0.65)
Mod HAS	97.1 (91.8–99.2)	17.9 (15.7–18.9)	34.0 (32.2–34.8)	93.3 (81.4–98.3)	1.23 (1.14–1.24)	0.05 (0.00–0.33)	0.71 (0.65–0.76)
AI Score	98.0 (93.1–99.7)	17.5 (15.4–18.2)	34.1 (32.4–34.7)	95.3 (83.6–99.2)	1.19 (1.10–1.22)	0.11 (0.02–0.45)	0.71 (0.65–0.76)

*A negative modified Heidelberg Appendicitis Score (HAS) or Artificial Intelligence (AI) almost rules out perforated appendicitis (NPV and negative LR). PPV, positive predictive value; NPV, negative predictive value; LR+, positive likelihood ratio; LR–L negative likelihood ratio; AUC, area under the curve*.

## Discussion

Different scoring systems have been published to aid diagnosing acute appendicitis. Among these scores, the Alvarado-, the Tzanakis, and the PAS have been validated most often. For these scores varying numbers of sensitivity (71.9–100%) and specificity (66.6–100%) were described in previous studies (14–17). Although in principal qualified to predict acute appendicitis, these scores lack clinical practicability due to their complexity. For instance, the Alvarado score and the PAS comprise of eight different features, which are weighed differently, complicating their applicability even further. The HAS comprises only of five features without any differentiated weighing and is therefore very easy to apply. Unfortunately, in the current study pain quality was rarely present. Additionally, it might be difficult to assess in younger children (18). On account of this considerable shortcoming, two new appendicitis scores were developed. Both scores outperform all previous scores by far with regards to their excellent sensitivity and decent specificity which will ensure clinical application.

The modified HAS and AI score strengthen the case for each other as they were generated by entirely different techniques but include almost the same items. They yield a nearly identical sensitivity and specificity. However, in contrast to all other scores, the AI based approach does not consider tenderness of the right lower quadrant to be a significant predictor of an acute appendicitis. It is for this unexpected finding, that we have serious doubts that this new AI score could find acceptance amongst physicians. Furthermore, tenderness of the lower right quadrant can be assessed very easily and quickly even by a physician without much experience and therefore is a very suitable indicator in the clinical practice. Furthermore, reproduction of the random forest-based determination of the important predictors of appendicitis is rather challenging for the reader or the intrigued clinician, whereas CART analysis is straight forward and can easily be reproduced and adjusted if desired. Therefore, we primarily suggest to promote the use of the modified HAS rather than the AI score.

In general, the modified HAS and AI score may suggest that clinical signs are less important than previously assumed in the diagnosis of pediatric appendicitis (19). This may be explained by atypical presentation of appendicitis in young children and limited communication skills in this age group (20).

Both new scores include CRP and WBC. They are non-specific markers of inflammation, whose diagnostic value regarding appendicitis have been evaluated numerously (21–24). Previous publications have stated a 100% negative predictive value for acute appendicitis, if both WBC and CRP are normal, whereas other reports found appendicitis in the presence of normal CRP, WBC, and neutrophils (22, 25). One recent study found the absence of neutrophilia to be the most important item to determine children with low risk of having appendicitis (26). Furthermore, in some publications, measurements of CRP and/or WBC revealed additional diagnostic value regarding the severity of appendicitis (e.g., advanced and perforated), whereas some publications negate such correlations (22, 23, 27). Some authors hypothesized that these confronting findings are the result of the different pathogenesis of simple and complicated appendicitis. Individual differences in immunity have been proposed as indicated by increased neutrophil and monocyte counts in complicated appendicitis and eosinophilia in simple appendicitis (28).

Both the original and the modified HAS include ultrasound (US) as an essential component. In our cohort, US demonstrating appendicitis seems to be one of the best predicting factors for appendicitis in children with abdominal pain. US has been used to aid diagnosing acute appendicitis since the 1980s and has already been evaluated extensively in pediatric populations (29–33). US is found to be the most cost-effective diagnostic approach in children with suspected appendicitis (34). The drawback of US is that its application can potentially delay surgical treatment and its diagnostic value is quite dependent on the experience of the user (29, 30, 32, 35). In this current and in previous studies, the embedding of US in the diagnostic algorithms or scoring system, improved sensitivity, and specificity (30). To some extent US outperforms clinical assessment of emergency physicians at diagnosing acute appendicitis (36).

### Limitations

Most limitations of the current study are inherent in a retrospective study. Furthermore, at both centers, nurse-directed triage pre-selected patients for the surgical department with a high probability of appendicitis which could affect the results of the current study limiting the generability of the two new scores. Hence, the two new scores should be evaluated in a cohort of patient with abdominal pain in order to validate our findings. Moreover, as in most studies that rely on clinical features, another limitation is the inter-observer variability, as experience may significantly affect the examiner's interpretation of the clinical findings (16). However, the modified HAS and the AI score should be very robust in this regard, as tenderness in the right lower quadrant and rebound tenderness are very basic factors which can be assessed easily. Ultimately, US is very user-dependent and in the absence of a specialized Pediatric Radiologist, trained Pediatrician, or Pediatric Surgeons are needed to assess the factors of the modified HAS. Since, the modifications of the score refer to the results in our cohort, prospective validation should be performed in future studies.

In summary, in our cohort all presented scoring systems are qualified to predict acute appendicitis with considerable restrictions regarding the clinical practicability. Since the modified Heidelberg Appendicitis and the Artificial Intelligence score demand the fewest clinical features without differentiated weighing, they provide the best clinical practicability while providing a good predictability for both appendicitis in general and perforated appendicitis. We recommend the modified HAS as it has excellent predictive capabilities, it is easy to assess and more likely be adopted by clinicians than the AI score which does include a key symptom of appendicitis. The modified HAS resembles the current diagnostic work up in most centers treating appendicitis in children. However, our findings should be validated in prospective, multicenter studies.

## Data Availability Statement

The original contributions generated in the study are included in the article/supplementary materials, further inquiries can be directed to the corresponding author.

## Ethics Statement

The studies involving human participants were reviewed and approved by Ethik-Kommission der Ärztekammer Hamburg, PV5459, and PV5891. Written informed consent from the participants' legal guardian/next of kin was not required to participate in this study in accordance with the national legislation and the institutional requirements.

## Author Contributions

CS and JE designed the study, collected data, analyzed the data, and drafted and revised the paper. MK, JH, C-MJ, TG, and KR collected data and drafted and revised the paper. MB designed the study, collected data, analyzed the data, performed statistics, and drafted and revised the paper. All authors contributed to the article and approved the submitted version.

## Conflict of Interest

The authors declare that the research was conducted in the absence of any commercial or financial relationships that could be construed as a potential conflict of interest.
